# Comparative immunodiagnostic evaluation of recombinant serine proteases from *Trichinella spiralis* and *Trichinella nativa* for early detection of trichinellosis

**DOI:** 10.14202/vetworld.2025.3243-3254

**Published:** 2025-10-31

**Authors:** Aibek Zhumalin, Aisarat Gajimuradova, Aitbay Bulashev, Orken Akibekov, Alfiya Syzdykova, Nurtai Gubaidullin, Fabio Tosini, Fariza Zhagipar, Ali Aydin, Nasipkhan Askarova

**Affiliations:** 1 Department of Veterinary Medicine, Faculty of Veterinary and Livestock Technology, S. Seifullin Kazakh Agrotechnical Research University, 62 Zhenis Avenue, Astana, 010011, Kazakhstan; 2 Organic Agrotechnology Engineering Center, Agrotechnical park Seifullin University, S. Seifullin Kazakh Agrotechnical Research University, 62 Zhenis Avenue, Astana, 010011, Kazakhstan; 3 Department of Microbiology and Biotechnology, Faculty of Veterinary and Livestock Technology, S. Seifullin Kazakh Agrotechnical Research University, 62 Zhenis Avenue, Astana, 010011, Kazakhstan; 4 European Union Reference Laboratory for Parasites, “lstituto Superiore di Sanità”, 299, Viale Regina Elena, Roma, 00161, Italy; 5 Division of Food Hygiene and Technology, Faculty of Veterinary Medicine, İstanbul University-Cerrahpasa, Üniversite MAhallesi, Üniversite Caddesi, 34320, Avcılar, İstanbul, Turkiye

**Keywords:** immunodiagnosis, recombinant antigens, serine protease, *Trichinella nativa*, *Trichinella spiralis*, trichinellosis

## Abstract

**Background and Aim:**

Trichinellosis, a major zoonotic foodborne disease caused by *Trichinella* spp., remains challenging to diagnose in its early stages due to low antibody titers and limitations of conventional excretory-secretory (ES) antigens. Recombinant antigens, particularly serine proteases expressed throughout infection, represent promising candidates for specific and sensitive diagnostics. This study aimed to clone, express, and evaluate recombinant serine proteases from *Trichinella spiralis* (rTsp-LE) to *Trichinella nativa* (rTnsp-4E), and to assess their diagnostic performance compared with ES antigens and a commercial kit.

**Materials and Methods:**

Serine protease genes of *T. spiralis* and *T. nativa* were amplified, cloned into pET28c+ vectors, expressed in *Escherichia coli*, and purified under denaturing conditions. Antigenicity was tested by immunoblotting and an indirect enzyme-linked immunosorbent assay using sera from experimentally infected rabbits, naturally infected pigs, and immunized BALB/c mice. Antibody dynamics were monitored over 30 day post-infection (dpi), and results were compared with ES antigens and the VetLine Trichinella kit. Statistical analyses were performed using one- and two-way analysis of variance.

**Results:**

Both recombinant proteins were successfully expressed at expected sizes (10.8 kDa for rTsp-LE; 6.8 kDa for rTnsp-4E) and induced strong, specific antibody responses. Antibodies against rTsp-LE and rTnsp-4E were detectable as early as 7 dpi, earlier than ES antigens (14–21 dpi). Immunized mice showed significant titers (up to 1:5700 for rTsp-LE and 1:10400 for rTnsp-4E by day 30). No cross-reactivity was observed with *Echinococcus* antigens. In rabbits, rTsp-LE showed the highest titers for *T. spiralis* infection, while rTnsp-4E was more specific for *T. nativa*. Overall sensitivity and specificity reached 100% and 88.2%, respectively.

**Conclusion:**

Recombinant serine proteases rTsp-LE and rTnsp-4E demonstrated high sensitivity, species-specific reactivity, and early antibody detection, outperforming ES antigens. These findings support their potential as reliable candidates for serological assays, contributing to earlier and more accurate trichinellosis diagnosis and improved epidemiological surveillance.

## INTRODUCTION

Trichinellosis, caused by parasites of the genus *Trichinella*, poses a significant zoonotic threat to both humans and animals. Globally, an estimated 11 million individuals are chronically infected with *Trichinella spiralis* [1, 2]. The genus comprises 10 recognized species and several unclassified genotypes, classified as encapsulated forms (*T. spiralis*, *Trichinella nativa*, *Trichinella britovi*) and non-encapsulated forms (*Trichinella pseudospiralis*, *Trichinella papuae*, and *Trichinella zimbabwensis*) [3]. Among these, *T. spiralis* is the most prevalent encapsulated species in temperate zones, while *T. nativa* circulates mainly among Arctic and subarctic carnivores, including those in northern Kazakhstan [4–7]. Human infections with *T. nativa* are relatively uncommon and are usually linked to the consumption of wild game meat in these regions [5].

The complex life cycle of *Trichinella* complicates early diagnosis, particularly during the intestinal phase, which precedes larval migration into muscle tissue. Transmission occurs through both sylvatic and domestic cycles, enabling larvae to invade multiple tissues and establish chronic infections [8]. Diagnostic methods conventionally rely on muscle biopsy and immunoassays using excretory-secretory antigens (ES-Ag, S-Ag). However, conventional enzyme-linked immunosorbent assay (ELISA) frequently yields false-negative results at early stage of infection and may cross-react with antigens from related parasites, thereby reducing diagnostic accuracy [9, 10].

To overcome these limitations, recombinant proteins are increasingly applied in trichinellosis diagnostics. For example, Han *et al*. [11] reported that rTsTryp-ELISA detects infection as early as 8 days post-infection (dpi), with 98.1% sensitivity and 98.7% specificity, outperforming ES-ELISA. Similarly, Grzelak *et al*. [1] demonstrated that recombinant *T. britovi* protein rTbES21, produced in *Pichia pastoris*, is a species-specific antigen with complete diagnostic specificity, enabling differentiation between *T. britovi* and *T. spiralis*. Hu *et al*. [12] identified the recombinant elastase-1 antigen of *T. spiralis*, which showed 97.4% sensitivity and 99.1% specificity and detected antibodies as early as 6–8 dpi. Nuamtanong *et al*. [13] further demonstrated that the recombinant serpin TsSERP exhibited excellent diagnostic value in pigs, with a sensitivity of up to 100% and specificity of 100% at later infection stages.

Serine proteases of *T. spiralis* are key virulence factors that mediate larval penetration of the intestinal epithelium and subsequent migration into muscle. These enzymes degrade host extracellular matrix proteins, facilitating tissue invasion [14, 15]. Gene silencing of serine proteases significantly impairs parasite invasion, development, and reproduction, underscoring their functional importance [16]. Recombinant serine proteases also elicit protective immune responses in mice, reducing adult worm burden and larval numbers, making them attractive candidates for vaccine and diagnostic development [17]. Sun *et al*. [18] confirmed the diagnostic value of a recombinant *T. spiralis* serine protease (TsSP), which achieved 98% sensitivity and 99% specificity, allowing infection detection as early as 19 dpi.

Akibekov *et al*. [19] evaluated the recombinant serine protease Tsp-LE of *T. spiralis*, demonstrating strong diagnostic performance, with ELISA sensitivity of 98%, specificity of 100%, and antibody detection as early as 14 dpi, without cross-reactivity to other parasites. These findings highlight its potential as a reliable marker for early serodiagnosis of trichinellosis.

Despite advances in molecular and immunological techniques, the early and accurate diagnosis of trichinellosis remains a major challenge. Conventional methods based on ES-Ag often show limited sensitivity during the intestinal stage of infection and are prone to cross-reactivity with antigens from other parasites, leading to false-negative or false-positive results. Although recombinant proteins have been explored as alternatives, most studies have focused primarily on *T. spiralis*, with relatively little attention given to *T. nativa*, a species of epidemiological importance in Arctic and subarctic ecosystems, including northern Kazakhstan. This knowledge gap limits the development of species-specific diagnostic assays capable of differentiating *T. spiralis* from *T. nativa*. Furthermore, while several recombinant antigens (RAs) such as trypsins, elastases, and serpins have shown promise, comprehensive comparative evaluations of recombinant serine proteases from multiple *Trichinella* species remain scarce. Such data are critical for improving diagnostic accuracy, understanding species-specific immune responses, and strengthening surveillance in both domestic and wildlife reservoirs.

This study was designed to obtain and comparatively evaluate recombinant serine protease antigens (RSPAs) from *T. spiralis* (rTsp-LE) and *T. nativa* (rTnsp-4E) to determine their diagnostic potential for trichinellosis. Specifically, the research aimed to: (i) Clone, express, and purify recombinant serine proteases in an *Escherichia coli* system; (ii) assess their antigenicity and ability to induce humoral immune responses in mice; (iii) compare their diagnostic performance with ES antigens and a commercial kit using sera from experimentally and naturally infected animals; and (iv) evaluate their species-specific reactivity and cross-reactivity profile. By addressing these objectives, the study seeks to provide foundational evidence for the use of rTsp-LE and rTnsp-4E as reliable, sensitive, and species-specific diagnostic markers, thereby contributing to the development of improved serological assays for the early detection and epidemiological monitoring of trichinellosis.

## MATERIALS AND METHODS

### Ethical approval

All stages of the research were conducted at the JSC “S” scientific and technical, facilities and at the Scientific and Production Platform for Agricultural Biotechnology, S. Seifullin Kazakh Agrotechnical Research University (KATRU). The Animal Ethics Committee of the Faculty of Veterinary Medicine and Animal Husbandry Technology, S. Seifullin KATRU, Astana, Kazakhstan, approved the care and use of laboratory animals (protocol No.1, February 23, 2023), in accordance with the Guidelines for the maintenance and care of animals (Interstate Standard, Government Standard 34088–2017) [20]. All procedures involving animals were conducted in compliance with high biosafety and animal welfare standards. All protocols were performed in accordance with the International Guidelines for Biomedical Research Involving Animals (International Guiding Principles).

### Study period and location

The study was conducted from April 2024 to February 2025 at the Scientific and Production Platform of Agricultural Biotechnology and the Joint Kazakh-Chinese Laboratory for Biological Safety-2, S. Seifullin KATRU.

### Sample collection and gene identification

*T. nativa* larvae isolated from the muscles of a spontaneously infected wolf (Akmolinskaya Region, Kurgaldzhinsky District, 2016) were used in this study [6]. *T. spiralis* larvae were kindly provided by Dr. Anne Mayer-Scholl, DVM, specialist in the Diagnostics, Genetics, and Pathogen Characterization Department at the Reference Center for Risk Assessment (BfR), Berlin, Germany. Molecular identification was performed using multiplex polymerase chain reaction (PCR) [6].

Propagation was performed in 7–8-month-old male Soviet Chinchilla rabbits weighing 4,400–4,600 g, which were infected with 2,500–3,000 *Trichinella* larvae. In each group, three rabbits were infected with *T. spiralis* and *T. nativa*. Subsequently, larvae were isolated from muscle tissue using the compression method [21].

The nucleotide sequences of the serine protease genes from the two *Trichinella* species were obtained and sequenced using the Sanger method (SeqStudio Genetic Analyzer, Applied Biosystems, USA) and analyzed using Basic Local Alignment Search Tool (BLAST), revealing 99.9% identity for both genes. The nucleotide sequences of the studied serine protease genes for *T. spiralis* (PP099881.1) and *T. nativa* (PP025901.1) were deposited in the National Center for Biotechnology Information (NCBI) database [19].

### RNA isolation and complementary DNA synthesis

Total RNA was isolated from five intestinal and five muscle larvae using TRIzol reagent (Invitrogen, USA) according to a standard protocol [22]. The RNA concentration was measured using NanoDrop 2000 (Thermo Scientific, USA) (average concentration, 209 ng/μL). The purity ratio (A260/A280) was 2.01, indicating high purity of the sample. cDNA was synthesized using the ProtoScript II First Strand cDNA Synthesis Kit (New England BioLabs, England) according to the manufacturer’s protocol. The cDNA product was stored at –20°C.

### PCR amplification, gene cloning, and transformation

Primers for molecular experiments were designed using Primer BLAST (NCBI) and tested in the Oligocalculator program (Berkeley, USA). The DNA sequences of the serine protease genes were reported in the National Center for Biotechnology Information database under accession numbers: PP099881.1 for *T. spiralis* and PP025901.1 for *T. nativa* [19].

The following primers were used for PCR amplification:

- *T. spiralis* F: 5’-AATCGTCTTGTGGATGCAAATGCAATAAC-3’
- *T. spiralis* R: 5’-CTCTAGTGAAACCCCAACCAGAAAGAAAAC-3’ (270 bp - 3 exon)
- *T. nativa* Pr4_F: 5’-GGCACGGAAAAACCAAGAG-3’
- *T. nativa* Pr4_R: 5’-GATTAGTAGAGTGCCGTTGC-3’ (156 bp - 4 exon).

PCR reactions were performed in a 20 µL mix containing 2 µL DreamTaq Buffer (Thermo Fisher Scientific, USA), 2.5 mM MgCl_2_, 0.8 mM dNTPs, 20 pmol of primer, 0.25 µL DreamTaq DNA polymerase (1 unit), and 100 ng cDNA.

PCR conditions for rTsp-LE (*T. spiralis*):

- 95°C for 3 min, then 30 cycles of 95°C for 30 s, 58°C for 30 s, 72°C for 1 min, followed by final extension at 72°C for 7 min.
- PCR conditions for rTnsp-4E (*T. nativa*):
- 95°C for 3 min, then 30 cycles of 95°C for 30 s, 63°C for 30 s, 72°C for 1 min, followed by final extension at 72°C for 7 min.

MQ water (3 μL) served as a negative control. Reactions were performed using a VeritiPro amplifier (Applied Biosystems, USA). Amplicons were analyzed by electrophoresis in 1% agarose gel with ethidium bromide [17] and extracted using the Monarch DNA Gel Extraction Kit (New England Biolabs, UK). Purified fragments were ligated into an expression vector using the CloneJET PCR Cloning Kit (Thermo Fisher Scientific, USA) in a 20 μL reaction volume. Transformation was performed using the heat-shock method in *E. coli* DH5α cells (NEB, UK) [18].

**Figure 1:**
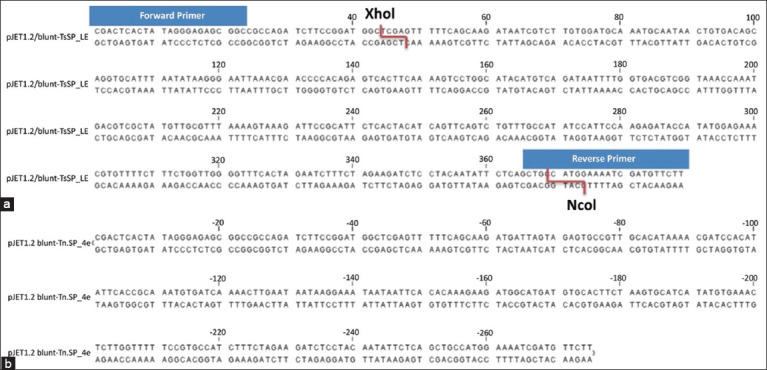
Schematic representation of recombinant clones for serine protease expression in the pJET2.1/blunt vector: (a) rTsp-LE from *Trichinella spiralis*; (b) rTnsp-4E from *Trichinella nativa* (CLC sequence Viewer 8.0 program).

Positive transformed colonies were selected based on resistance to ampicillin (50 μg/mL). Recombinant plasmids were purified using the Monarch Plasmid Miniprep Kit (New England Biolabs, UK). The genes were excised using NcoI and XhoI restriction sites (Thermo Fisher, USA) for insertion into the pET28c+ expression vector (Figure 1). Colonies transformed into *E. coli* DH5α with the pET28c/Tsp-LE and pET28c/Tnsp-4E expression constructs were selected on LB medium containing kanamycin (50 μg/mL). Vector integration was confirmed by Sanger sequencing on a SeqStudio Genetic Analyzer (Applied Biosystems, USA).

### Protein induction and purification

Overnight cultures (5 mL) from positive clones were transferred into 500 mL LB broth containing kanamycin (50 µg/mL). Cultures were incubated at 37°C with 5.41 × *g* until OD_600_ = 0.6, then induced with 0.2–0.5 mM Isopropyl−β-D-1-thiogalactopyranoside [IPTG] for 5 h.

Cells were harvested by centrifugation at 9,000 × *g* for 1 h at 4°C, resuspended in lysis buffer (50 mM Tris-HCl, 500 mM NaCl, 1% Triton X-100, 1 mM phenylmethylsulfonyl fluoride [PMSF], 5 mM β-mercaptoethanol, pH 8.0), and lysed by ultrasonication (5 × 30 s bursts at 80 GHz, with 2 min cooling intervals). Inclusion bodies were collected at 15,000 × *g*, resuspended in lysis buffer, centrifuged again, and solubilized in denaturing buffer (phosphate-buffered saline [PBS], 8 M urea, 2 mM ethylenediaminetetraacetic acid, 1 mM PMSF) for 1 h at 4.47 × *g* [23]. Clarified lysates were processed by fast protein liquid chromatography using a HisTrap HP column (Cytiva, USA) on an ÄKTA Pure 25 system (Cytiva, USA). Fractions above 300 mAU were pooled, and yields averaged 11–12 µg/L. Proteins were stored at –20°C.

### Immunization of mice and antibody titration

Male mice (5–7 weeks, 20–25 g) were divided into four groups of three each. Animals were maintained at 20°C–22°C with 45%–60% humidity and fed a balanced commercial diet (Little One, Mealberry, Russia). Group I received rTsp-LE, Group II received rTnsp-4E, and the control group received PBS.

Immunization consisted of five intraperitoneal injections (50 µg/mL; 100 µL/dose). On days 1 and 7, antigens were emulsified 1:1 with Freund’s complete or incomplete adjuvant (Thermo Fisher Scientific, USA). Boosters were given on days 11, 12, and 13 with PBS-dissolved antigens.

Blood samples were collected on days 10 and 17. Antibody titers were determined by endpoint ELISA using immobilized RAs and ES-Ag (0.005 mg/mL), with a VetLine Trichinella kit (NovaTec Immunodiagnostica GmbH, Germany).

### Experimental infection of mice

Three-month-old mice were divided into experimental (n = 18) and control (n = 3) groups. Each experimental group was orally infected with 250–300 *T. spiralis* or *T. nativa* larvae derived from infected rabbits. Blood samples were collected from the tail vein at 3, 5, 7, 14, 21, and 30 dpi.

### Immunoassay and antigen characterization

Antibody titers were measured by indirect ELISA with RAs rTsp-LE and rTnsp-4E (0.005 mg/mL) [24]. Plates were incubated overnight at 4°C, blocked, and probed with sera diluted 1:100. Binding was detected using anti-mouse immunoglobulin G (IgG) (Sigma, USA; 1:10,000) and Tetramethylbenzidine [TMB] substrate (Immunotech, Russia). Absorbance was measured at 450 nm (BioSan, Latvia).

For immunoblotting, bacterial lysates and purified proteins were separated by sodium dodecyl sulfate-polyacrylamide gel electrophoresis, transferred to nitrocellulose membranes (Bio-Rad, USA), and probed with primary sera (1:100) [25]. Secondary detection used alkaline phosphatase-conjugated goat anti-rabbit IgG (Sigma-Aldrich, USA; 1:30,000). Visualization employed 5-Bromo-4-Chloro-3-Indolyl phosphate [BCIP] substrate, and bands were imaged using a GelDoc Go System (Bio-Rad, USA) [26].

Positive control sera were obtained from *Trichinella*-infected swine (Prof. Karsten Nöckler, BfR). Negative controls were collected from 74 pigs in the Akmola region. Two additional positive sera were collected from experimentally infected rabbits in this study.

### Statistical analysis

All statistical analyses were performed using GraphPad Prism v8.0.1 (GraphPad Software, USA). Results are presented as mean ± standard deviation. Each experiment included at least three biological replicates. Differences between groups were analyzed using one-way analysis of variance (ANOVA). Where multiple factors were involved, two-way ANOVA was used. A p < 0.05 was considered statistically significant.

## RESULTS

### Cloning of RAs in an *E. coli* expression vector

**Figure 2:**
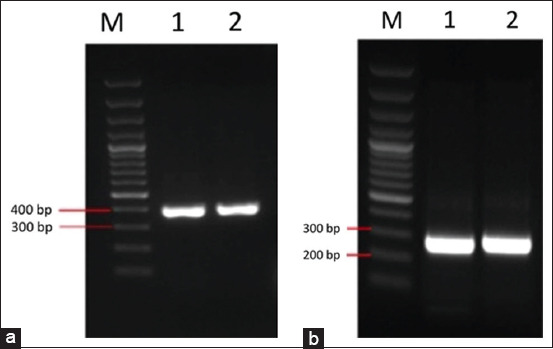
Polymerase chain reaction result with serine protease gene of *Trichinella spiralis* (399 bp) (a) and *Trichinella nativa* (254 bp) (b) (product size indicated with restriction sites).

To obtain DNA fragments encoding rTsp-LE from *T. spiralis* and rTnsp-4E from *T. nativa*, total RNA was extracted from intestinal larvae and retro-transcribed to produce cDNA as a template for PCR amplification. The DNA amplicons for rTsp-LE and rTnsp-4E were 399 and 254 bp, respectively (Figure 2).

### Construction of expression vectors and clone verification

The amplified DNA fragments were directly cloned into pJET1.2/blunt and replicated in *E. coli* cells. The recombinant inserts were excised by restriction digestion from pJET1.2/blunt-derived plasmids and ligated into the linearized pET-28 c+ to obtain the final expression vectors to produce the RAs. Recombinant clones were selected by PCR screening of bacterial colonies after transformation with ligated plasmids. Positive clones were confirmed by Sanger sequencing in both the first vector pJET1.2/blunt and pET-28 c+ vector. Sequencing of rTsp-LE and rTnsp-4E positive clones in pET-28 c+ cells also confirmed the proper insertion of recombinant fragments in-frame with histidine tags at the end of the *Trichinella* antigens.

### Expression and purification of recombinant proteins

**Figure 3:**
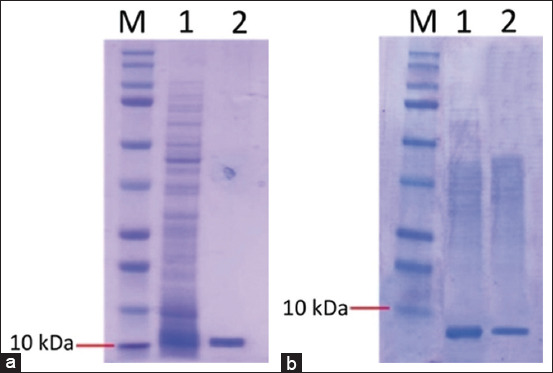
Purification of recombinant peptides by affinity chromatography (Ni^2+^-sorbing column): (a) Purification of rTsp-LE: 1 - clarified lysate loaded on the column, 2 - purified fraction eluted from the column; (b) purification of rTnsp-4E: 1 - clarified lysate loaded on the column, 2 - purified fraction eluted from the column. M-protein ladder (Biorad, USA, 10–250 kDa).

Two positive clones, one for rTsp-LE expression and one for rTnsp-4E expression, were induced to express the recombinant peptides, and the tagged proteins were purified by affinity chromatography as described in the Materials and Methods section. The expected molecular weights of the two recombinant products were 10.8 kDa for rTsp-LE and 6.8 kDa for rTnsp-4E, and the expected protein bands were perfectly consistent with the observed products in the protein gel (Figure 3). Recombinant TsSP protein was obtained at a concentration of 410 μg/mL and *T. nativa* at 450 μg/mL.

### Immunochemical characterization by immunoblotting

**Figure 4:**
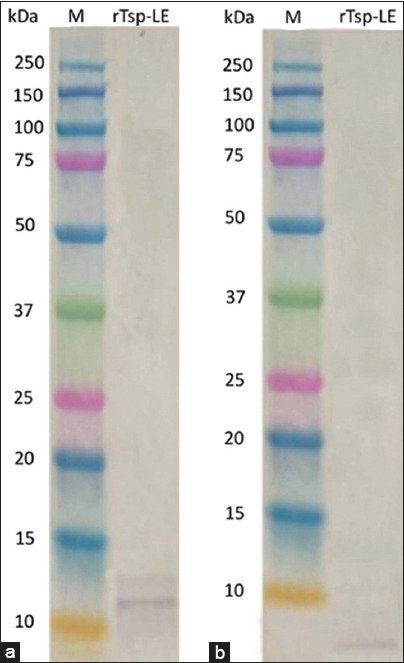
Immunoblotting of recombinant rTsp-LE from *Trichinella spiralis* and rTnsp-4E from *Trichinella nativa* proteins recognized by the serum of an experimentally infected rabbit, M - molecular weights marker (Biorad, USA, 10–250 kDa).

The two RAs were initially characterized by immunoblotting using the positive serum of experimentally infected rabbits. The results are shown in Figure 4, and the rabbit serum recognized both the purified antigens, labeling a band larger than 10 kDa in the Tsp-LE electrophoretic lane and a band smaller than 10 kDa in the rTnsp-4E electrophoretic lane. Overall, these data confirmed the responsiveness of the rabbit immune system to these two antigens during a *Trichinella* infection.

### Immunization-based antigenicity assessment (ELISA, Day 10 and Day 17)

The antigenicity of the two antigens was further tested by immunizing mice with these antigens. The dynamics of antibody production in mice were assayed by enzyme-linked immunosorbent assay on days 10 and 17 after immunization. RAs rTsp-LE and rTnsp-4E, as well as ES-Ag as a control, were used for immobilization. Table 1 presents the results of the assay. Immunoenzymatic analysis of serum antibodies demonstrated a consistent increase in titers against both recombinant proteins during the immunization period. In group I, a positive antibody titer against the homologous antigen rTsp-LE was detected on the 10^th^ day after immunization (1:300). On the 17^th^ day, the titer increased 13-fold compared with the initial level. In contrast, the response against the heterologous antigen rTnsp-4E was negative on the 10^th^ day and reached only 1:300 on the 17^th^ day, which was considered positive but remained markedly lower than the homologous response. These results confirmed the antigenic specificity of rTsp-LE for *T. spiralis* detection. In group II, immunized with rTnsp-4E, a positive antibody response was observed as early as 10^th^ day (1:490), which further increased on 17^th^ day (1:4850). In this group, the response against rTsp-LE was negative on the 10^th^ (1:120) but became positive on the 17^th^ (1:610), confirming the antigenic specificity of rTnsp-4E. Analysis against ES-Ag revealed positive titers for both RAs only on the 17^th^ day, while results at 10^th^ remained negative. No cross-reactivity was recorded with the *Echinococcus* ES-Ag antigen, except for a weak response (1:100) on the 17^th^ day. No antibody titers were detected in the control group at either time point (10^th^ and 17^th^ day), indicating the absence of nonspecific reactions. Overall, immunization with the rTsp-LE and rTnsp-4E recombinant protein antigens induced effective humoral responses and promoted the production of highly specific antibodies.

**Table 1:** Dynamics of antibody production in the sera of immunized rTsp-LE and rTnsp-4E mice.

Animal groups	rTsp-LE		rTnsp-4E	
	
	Days after immunization			

	10^th^	17^th^	10^th^	17^th^

	Average antibody titer			
I group *rTsp-LE*	1:300 (+23,1; −18,7)	1:3940 (+23,1; −18,7)	1:100 (+23,1; −18,7)	1:300 (+23,1; −18,7)
II group *rTnsp-4E*	1:120 (+23,1; −18,7)	1:610 (+23,1; −18,7)	1:490 (+23,1; −18,7)	1:4850 (+23,1; −18,7)
Control group	Negative	Negative	Negative	Negative

**Animal groups**	**ES-Ag**		**ES-Ag *Echinococcosis***	
	
	**Days after immunization**			

	**10^th^**	**17^th^**	**10^th^**	**17^th^**

	**Average antibody titer**			

I group *rTsp-LE*	1:250 (+23,1; −18,7)	1:1970 (+60,2; −37,5)	Negative	1:100 (+23,1; −18,7)
II group *rTnsp-4E*	1:200 (+23,1; −18,7)	1:2430 (+23,1; −18,7)	Negative	Negative
Control group	Negative	Negative	Negative	Negative

ES-Ag = Excretory-secretory antigens.

### Serological kinetics in experimentally infected mice (Up to 30 dpi)

At each stage of infection, serum was collected from the mice for antibody testing by indirect enzyme-linked immunosorbent assay against the obtained RAs and ES-Ag. The results are summarized in Table 2. Immunoactive characterization of antigens was performed using serum from experimentally infected mice. The mean antibody titers in mice, measured using the rTsp-LE and rTnsp-4E RAs, demonstrated a dynamic and specific humoral response over 30 dpi. In Group I (against rTsp-LE), antibodies to the homologous antigen were detectable by 7 dpi (1:250) and gradually increased to 1:5700 by 30 dpi. At the same time, the cross-reaction to rTnsp-4E (1:120–1:4850). In Group II (against rTnsp-4E), an early positive response to the homologous antigen was recorded on 7 dpi (1:300), peaking at 1:10400 by 30 dpi. At the same time, the cross-reaction to rTsp-LE was moderate (1:100–1:9700). Analysis using the ES-Ag antigen showed the formation of antibodies: In Group I, the first titers appeared on 14 dpi (1:200), reaching 1:7880 by 30 dpi, and in Group II, titers appeared on 21 dpi (1:980) and rose to 1:9700 by 30 dpi. Comparison with the commercial VetLine *Trichinella* kit (NovaTec Immunodiagnostica GmbH, Germany) showed a similar titer dynamic and high correlation with the ES-Ag results, with only minor differences observed at later stages. No antibodies were detected in the control group, confirming the absence of non-specific reactions. Therefore, the RAs rTsp-LE and rTnsp-4E induce a strong, specific humoral response and enable the detection of antibodies against *T. spiralis* and *T. nativa* at both early and late stages of the immune response, comparable to the commercial VetLine kit (NovaTec Immunodiagnostica GmbH).

**Table 2:** Mean antibody titers of *Trichinella* larvae-infested mice.

Animal groups/days after the invasion	Average titers of antibodies to the rTsp-LE antigen					

	3 (dpi)	5 (dpi)	7 (dpi)	14 (dpi)	21 (dpi)	30 (dpi)
Group I rTsp-LE	PO	PO	1:250 (+26.6; −21.1)	1:610 (+26.6; −21.1)	1:2600 (+26.6; −21.1)	1:5700 (+26.6; −21.1)
II rTnsp-4E group	PO	PO	1:100 (+23.1; −18.7)	1:250 (+26.6; −21.1)	1:1970 (+26.6; −21.1)	1:9700 (+26.6; −21.1)
Control group	PO	PO	PO	PO	PO	PO
Average titers of antibodies to the rTnsp-4E antigen						
Group I rTsp-LE	PO	PO	1:120 (+23.1; −18.7)	1:490 (+23.1; −18.7)	1:1600 (+26.6; −21.1)	1:4850 (+23.1; −18.7)
II rTnsp-4E group	PO	PO	1:300 (+23.1; −18.7)	1:570 (+26.6; −21.1)	1:2430 (+23.1; −18.7)	1:10400 (+23.1; −18.7)
Control group	PO	PO	PO	PO	PO	PO
Average titers of antibodies to ES-Ag						
I group	PO	PO	PO	1:200 (+26.6; −21.1)	1:1210 (+23.1; −18.7)	1:7880 (+26.6; −21.1)
II group	PO	PO	PO	PO	1:980 (+26.6; −21.1)	1:9700 (+23.1; −18.7)
Control group	PO	PO	PO	PO	PO	PO
Average titers of VetLine *Trichinella* antibodies						
Group I rTsp-LE	PO	PO	1:250 (+26.6; −21.1)	1:400 (+23.1; −18.7)	1:1210 (+26.6; −21.1)	1:7880 (+23.1; −18.7)
II rTnsp-4E group	PO	PO	1:200 (+23.1; −18.7)	1:300 (+26.6; −21.1)	1:1970 (+23.1; −18.7)	1:6400 (+23.1; −18.7)
Control group	PO	PO	PO	PO	PO	PO

ES-Ag = Excretory-secretory antigens, dpi = Days post-infection.

### Diagnostic evaluation in rabbits and pigs (comparative antigen performance)

The RAs were also tested with animal sera from experimentally infected rabbits and from naturally infected pigs and compared with ES-Ag and commercial kits. Table 3 summarizes the results of these experiments. The RAs rTsp-LE showed maximum titers in *T. spiralis*-infected rabbits (1:10400), reflecting a species-specific immune response. The level was lower in animals infected with *T. nativa* (1:7350) and in the positive swine serum, it was even lower (1:3680). This pattern suggests cross-reactivity but points to this antigen’s limited universality. Conversely, the rTnsp-4E antigen titers showed the opposite pattern: the highest titers were observed in rabbits infected with *T. nativa* (1:9700). In contrast, titers for *T. spiralis* remained significant (1:8440). The antibody levels in the swine sera were minimal (1:3200), emphasizing the specificity of the antigen for *T. nativa* diagnosis. ES-Ag yielded more balanced results, providing comparable titers in both rabbit groups (*T. spiralis*: 1:7880 and *T. nativa*: 1:7350). Furthermore, the reactivity in the swine serum (1:3940) was higher than that observed with the RAs. This indicates the greater universality of ES-Ag and its suitability for broad application. The results of the commercial VetLine kit were comparable to those of the ES-Ag, with identical metrics. This confirms the use of similar antigenic components and underscores the reliability of the kit for routine trichinellosis diagnosis.

**Table 3:** *Trichinella*-specific antibody titers compared by limit dilution against different antigenic substrates (ES-Ag, rTsp-LE, and rTnsp-4E) from the sera of experimentally and naturally infected rabbits and pigs.

Antigens	The average antibody titer		

	*Trichinella spiralis* infection in rabbits (45 dpi)	*Trichinella nativa*-infected rabbits (45 dpi)	Positive *Trichinella spiralis* pig serum
rTsp-LE	1:10400 (+23.1; −18.7)	1:7350 (+14.9; −13.0)	1:3680 (+41.4; −29.3)
*rTnsp-4E*	1:8440 (+14.9; −13.0)	1:9700 (+23.1; −18.7)	1:3200 (+32.0; −24.2)
ES-Ag	1:7880 (+23.1; −18.7)	1:7350 (+23.1; −18.7)	1:3940 (+39.5; −28.2)
Commercial VetLine kit	1:7880 (+23.1; −18.7)	1:7880 (+23.1; −18.7)	1:3940 (+39.5; −28.2)

ES-Ag = Excretory-secretory antigens, dpi = Days post-infection.

## DISCUSSION

### Zoonotic significance of *Trichinella* infections

Roundworms of the genus *Trichinella* remain a major zoonotic foodborne concern, with *T. spiralis* and *T. nativa* being the most widespread species responsible for human infections [27]. Early diagnosis is still difficult because of low antibody levels during the 1^st^ week of infection [28].

### Limitations of conventional diagnostic approaches

Conventional antigens, such as ES-Ag and S-Ag, are labor-intensive to obtain and prone to cross-reactions [29, 30], necessitating ELISA confirmation by Western blotting in line with International Commission on Trichinellosis guidelines [31]. These challenges highlight the need for more reliable, standardized diagnostic tools.

### Advantages of RAs

In this context, RAs provide a cost-effective, reproducible, and highly specific alternative, offering clear advantages for routine veterinary surveillance [1, 12]. This study demonstrated that the RAs rTsp-LE and rTnsp-4E could elicit strong, highly specific antibody responses, confirming their potential for trichinellosis serodiagnosis. The distinct reactivity patterns observed between homologous and heterologous antigens underscore their species-specific nature, which is essential for the differential diagnosis of *T. spiralis* and *T. nativa*. Importantly, the absence of significant cross-reactivity with heterologous helminths indicates that these antigens provide a high level of diagnostic reliability compared with crude preparations. Together, these findings support the use of RSPAs as promising components of improved diagnostic platforms, which may enhance both early detection and epidemiological surveillance of *Trichinella* infections.

### Role of serine proteases in pathogenesis and diagnosis

Serine proteases are the most abundant proteinases in the excretory-secretory products of *Trichinella* spp. and are key molecules in parasite invasion [32–35]. Their high expression during the intestinal infective larval stage and detection of stage-specific antibodies emphasize their role as early diagnostic markers [36, 37]. In addition, TspSP-1.3 was abundantly expressed across several life stages, identified in ES products, and its recombinant form reduced muscle larval burden in mice by 39% [17]. These results support its relevance as both a diagnostic antigen and a potential vaccine target.

### Study findings and diagnostic performance

In this study, the RAs rTsp-LE (*T. spiralis*) and rTnsp-4E (*T. nativa*) demonstrated strong immunogenicity and species-specific reactivity. rTsp-LE showed the highest titers in *T. spiralis*-infected rabbits (1:10400), whereas rTnsp-4E was most reactive with *T. nativa* sera (1:9700). Both antigens exhibited lower responses in pig sera, supporting their specificity. In contrast, ES-Ag and the VetLine kit (NovaTec Immunodiagnostica GmbH) showed balanced reactivity across both *Trichinella* species and greater sensitivity with pig sera, confirming their value for broad-scale screening. Importantly, no significant cross-reactivity with heterologous helminths was observed, highlighting the diagnostic reliability of recombinant proteins. The results showed 100% sensitivity and 88.2% specificity.

### Implications for diagnostic strategies

Taken together, our findings showed that rTsp-LE and rTnsp-4E are promising candidates for species-specific serodiagnosis of *T. spiralis* and *T. nativa*, whereas ES-Ag and VetLine remain suitable for routine screening. A combined strategy using RAs for precise identification and universal antigens for large-scale surveillance appears to be optimal for strengthening meat safety control and trichinellosis monitoring. Future work should include wider validation across host species and the development of multi-antigen ELISA platforms to improve sensitivity and specificity in field conditions.

### One Health approach

Trichinellosis exemplifies a classic One Health challenge, where human health, animal health, and food safety intersect. Transmission occurs through the consumption of undercooked or contaminated meat, linking livestock management, wildlife reservoirs, and human practices. By incorporating species-specific RAs into diagnostic platforms, early detection can be improved not only in livestock and wild animals but also in food inspection systems. This approach supports integrated control strategies that reduce the zoonotic risk, safeguard consumer health, and protect livestock productivity. A One Health-oriented diagnostic framework, combining veterinary surveillance, food chain monitoring, and public health interventions, is therefore essential to mitigate the burden of trichinellosis.

## CONCLUSION

This study successfully demonstrated that RSPAs rTsp-LE (*T. spiralis*) and rTnsp-4E (*T. nativa*), elicit strong, specific, and early antibody responses, making them promising candidates for species-specific serodiagnosis of trichinellosis. The homologous reactivity of rTsp-LE with *T. spiralis* sera (titers up to 1:10400) and rTnsp-4E with *T. nativa* sera (1:9700) highlights their diagnostic specificity, while minimal cross-reactivity with heterologous helminths underscores their reliability. In contrast, ES-Ag and the VetLine kit produced balanced results across species, reflecting their suitability for broad-scale surveillance. Overall, the recombinant proteins achieved 100% sensitivity and 88.2% specificity, outperforming crude antigen preparations, especially during the early stages of infection.

From a practical perspective, these findings provide strong evidence for integrating RAs into diagnostic platforms for veterinary and public health applications. The species-specific reactivity of rTsp-LE and rTnsp-4E is particularly valuable for distinguishing *T. spiralis* from *T. nativa* infections, an important feature for epidemiological mapping and risk assessment in regions where both species circulate. A combined diagnostic strategy, using RAs for species-level identification alongside ES-Ag for broad screening, offers an effective framework for improving food safety, animal health, and human health surveillance.

The strengths of this study lie in its comprehensive evaluation of RAs across multiple hosts (mice, rabbits, and pigs), confirmation of species-specific responses, and comparison with established diagnostic tools. However, limitations include the relatively small number of tested field sera and the restricted range of host species, which may affect the generalizability of findings. In addition, while strong humoral responses were observed, the potential of these antigens in field-based rapid tests remains unexplored.

Looking to the future, validation of rTsp-LE and rTnsp-4E in larger field populations, across diverse geographic regions and animal reservoirs, will be essential. The development of multiplex or multi-antigen ELISA platforms incorporating these RAs could enhance sensitivity and specificity under field conditions. Furthermore, given the immunogenicity of serine proteases, their role as vaccine candidates merits exploration to complement diagnostic applications.

In conclusion, rTsp-LE and rTnsp-4E represent robust, species-specific antigens with strong potential to advance the early and reliable diagnosis of trichinellosis. By combining recombinant tools with existing diagnostic strategies in a One Health framework, this work contributes to improved surveillance, safer meat production, and reduced zoonotic transmission risk.

## DATA AVAILABILITY

The datasets generated and analyzed during the present study are available from the corresponding author.

## AUTHORS’ CONTRIBUTIONS

AZ: Primer design and molecular analysis. AG: Drafted the manuscript and statistical analysis. OA: Experiment design, general management, and drafted the manuscript. AB: General management and drafted the manuscript. AS: Immunoactivity and immunodiagnostic experiments. NG: Bioinformatics analysis and protein typing experiments. FT: Analyzed the data and drafted the manuscript. FZ: Analyzed the data and wrote, reviewed, and edited the manuscript. AA: Experiment design and statistical analysis. NA: Experiments on obtaining genetically engineered constructs. All authors have read and approved the final manuscript.
